# *Self Portrait With Dr. Arrieta* and the Medical Profession's Tenuous Status in the Public Eye

**DOI:** 10.1093/ofid/ofu077

**Published:** 2014-12-23

**Authors:** Joel T. Katz, Shahram Khoshbin

**Affiliations:** 1 Division of Infectious Diseases; 2 Department of Neurology, Brigham and Women's Hospital, Harvard Medical School, Boston, Massachusetts

Works by Francisco José de Goya y Lucientes (1746–1828) bridge the gulf in many ways, from the 18th-century European masters to the early seeds of modernism. In the wake of the revolution to the north and the Napoleonic Wars during his lifetime, tumultuous political and social upheavals in Spain impacted the artist, directly. Goya's artistic opus transitioned from that of the celebrated chief court painter for King Charles IV (later shunned by his successor, Ferdinand VII) to his elderly personal works, focused on common life scenes, popular events, and depictions of “capricious subjects” (witches, ghosts, and monsters). Evidence suggests that his failing health may have played a central role; the fulcrum for this transition occurred in proximity to Goya's first known health crisis. As frequently occurs for true innovators, Goya's great genius was not fully appreciated until long after his death, which occurred in isolation and ignominy.

At age 46, the painter experienced a severe febrile illness while traveling in Cadiz that led to significant disability and the first of a number of periods of medical convalescence. Although the diagnosis of Goya's illness remains unproven [1], exposure to syphilis seems likely, and many of his symptoms parallel the ravaging sequential stages caused by this spirochete [2]. He ultimately developed what would now be called congestive heart failure as well as permanent deafness.

In *Self-Portrait with Dr. Arrieta* (1820), the cynical Goya offers a sentimental portrayal of his personal physician, Dr. Eugenio Garcia Arrieta [3, 4]. The subject on the right is Goya—the last known of his roughly 40 self-portraits—who appears vulnerable, gloomy, ghost-like, and faintly clinging to a cloth representing life. Arrieta, on the left, appears selfless, attentive, and fully supportive; his positioning keeps Goya erect and also the dark (possibly afterworld) characters behind him at bay. The artist adds a grateful inscription at the bottom: “Goya in gratitude to his friend Arrieta for the skill and great care with which he saved his life in his acute and dangerous illness, suffered at the end of 1819.”[Fig OFU077F1]

**Figure 1. OFU077F1:**
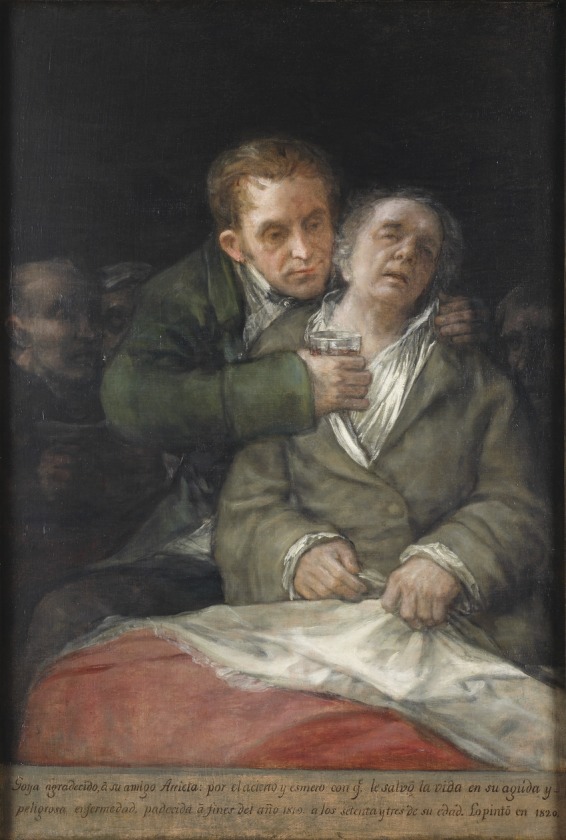
Cover: Francisco José de Goya y Lucientes. “Self Portrait with Dr. Arrieta.” Oil on canvas, 1820. Minneapolis Institute of Arts, Minnesota.

A careful look at this rich image offers insight into Goya's state and also into issues that are quite relevant to the current 21st century social discourse. It is ironic and informative to see the distain with which Goya held the medical profession (see, “Of What Ill Will He Die?”, an etching in which the doctor is portrayed as an ass [5]) and, at the same time, the deep admiration he demonstrated towards his own physician. Surveys about the modern healthcare system suggest widespread public distrust of the medical profession and negative impressions of physicians-at-large, yet high numbers of individuals who admire their own doctors. Two hundred years later, how is it that the profession is still distrusted, yet the individual practitioners are held in high esteem?
